# Editorial: Protein nanoparticles: characterization and pharmaceutical application

**DOI:** 10.3389/fphar.2023.1229068

**Published:** 2023-06-27

**Authors:** Agnese Gagliardi, Juan M. Irache, Donato Cosco

**Affiliations:** ^1^ Department of Health Sciences, University “Magna Græcia” of Catanzaro, Campus Universitario “S. Venuta”, Catanzaro, Italy; ^2^ Department of Chemistry and Pharmaceutical Technology, University of Navarra, Pamplona, Spain

**Keywords:** proteins, nanoparticles, drug delivery, biopolymers, pharmaceutical technology

The use of proteins as suitable biomaterials for pharmaceutical applications is a well-known strategy to modulate the physico-chemical and biopharmaceutical features of the active compounds (Martínez-López et al., 2020; Can Karaca et al., 2023). The specific structural properties of proteins can promote the development of various formulations such as micro/nanoparticles, scaffolds, (hydro)gels and films, offering a wide range of administration routes for the entrapped compound(s) (Elzoghby et al., 2012; Aljabali et al., 2022). A milestone of the use of protein for drug delivery purposes is represented by human albumin exploited for the development of nanoparticles containing paclitaxel (Spada et al., 2021). This nanoformualtion avoided the use of toxic organic solvents for the administration of the lipophilic compound, increased the drug localization within various solid tumors and today it is used in several clinical protocols (Kianfar, 2021).

Besides the animal proteins, the vegetal derivatives demonstrated to be versatile biopolymers that can be proposed as materials to obtain novel pharmaceutical formulations (Tran et al., 2019; Voci et al., 2021). For example, the family of prolamines has been widely investigated due to the biocompatibility and biodegradability, the wide availability on the market (also as waste materials of industrial processes) and the opportunity to easily modified the structure of its members (Elzoghby,et al., 2017; Voci et al., 2020).

This Research Topic is a collection of articles describing peculiar aspects of the use of proteins as drug carriers. The research articles discuss the *in vivo* toxicity of sericin from silk worm and the efficacy of formulations made up of the animal protein in cell regeneration. Other articles are focused on the state of the art of zein and silk fibroin as biomaterials for drug delivery applications.

Liu et al. focused on the description of zein, the major storage protein of corn, as natural biomaterial for pharmaceutical applications. They discussed the various preparation methods proposed to obtain zein nanoparticles, the physico-chemical parameters investigated in the phases of preformulation and the ways to evaluate the entrapment efficiency of bioactives. In addition, the authors described the application of zein nanosystems to improve the storage stability of the entrapped compounds, to increase their oral bioavailabilty, to obtain a controlled release of cargo molecules and to enhance the drug targeting. Finally, an analysis of the main criticisms compromising a translation of the most promising formulations from the bench to the market was provided.

Yu et al. evaluated the sate of the art of natural silk fibroin as biomaterial to be used for drug delivery purposes in chemotherapy. Useful information about the structure, chemical properties, mechanical resistance and biodegradability of the proteiun was provided. Moreover, the rationale of using silk fiborin as main component of drug delivery systems for antitumor application was discussed; in detail, the development of micro/nanosystems contining various active compounds and their efficacy against several solid tumors (i.e., breast, liver, lung, gastric and pancreatic carcinoma) have been reported. The authors also discussed the drawbacks related to the approval by the FDA of various fibroin systems for human application.

The teratogenic activity on pregnant rats of another protein extracted from silk, sericin, was investigated by Li et al. The obtained results demonstrated that the body weights, food consumption, and food utilization rates of pregnant rats were not affected by the oral administration of sericin up to a protein concentration of 1 g/kg. Moreover, the body weight, body and tail lengths of fetuses of rats treated with sericin were similar to those of the negative control group. In addition, no malformations of fetuses have been detected. This study confirmed the suitability of the animal protein to be used as biomaterial for oral administration because characterized by significant safety also when high concnetrations are administered.

The same protein was used by Bari et al. to obtain micro/nanoparticles containing crocetin proposed for the protection of nucleus pulposus cells from damage induced by oxidative stress. “Active per sé” nano-in micro formulations have been developed spray-drying the sericin/crocetin nanoparticles. These formulations demonstrated to be characterized by significant antioxidant, anti-elastase and anti-tyrosinase activities and to effectively avoid the toxicity against the cells of nucleus pulposus when an oxidative stress is applied.

In summary, the articles in this Research Topic provide some examples of the potential impact that proteins can exert in the pharmaceutical field and in the treatment of various diseases. Although the clinical use of protein-based systems is still in infancy, the Guest Editors opine that these biomaterials represent valuable natural compounds to develop several innovative formulations to be used on humans.
